# Characteristics influencing acute laryngitis and laryngeal obstruction in children: A retrospective study

**DOI:** 10.1097/MD.0000000000040885

**Published:** 2024-12-27

**Authors:** Yueting Zhu, Xuyuan Sun, Guangping Liu

**Affiliations:** a Department of Otolaryngology Head and Neck Surgery, Tianjin Children’s Hospital (Children’s Hospital of Tianjin University), Tianjin, China.

**Keywords:** children, influencing factors, laryngeal stenosis, laryngitis, signs and symptoms

## Abstract

Acute laryngitis lesions without appropriate management can lead to laryngeal obstruction, which is a life-threatening disease in children. This study analyzes the clinical characteristics that influence laryngeal obstruction in children with acute laryngitis. A total of 390 children who were treated in the hospital from February 2021 to February 2024 were retrospectively divided into 5 groups according to the degree of laryngeal obstruction (no, I°, II°, III°, and IV° laryngeal obstructions). The higher the degree of obstruction, the more significant the symptoms between the subgroups (*P* < .05). The sore throat, hoarseness, wheezing, barking cough, dyspnea, diffuse congestion and swelling of the laryngeal mucosa, and vocal cord congestion are most significant symptoms. The levels of biochemical markers in the IV° laryngeal obstruction group were higher than in the other 4 groups (*P* < .05). Multivariate logistic regression analysis showed that the tumor necrosis factor-alpha, interleukin (IL)-4, IL-6, IL-17, and interferon-gamma levels were risk factors for acute laryngitis complicated by laryngeal obstruction. Tumor necrosis factor-alpha, IL-4, IL-6, IL-17, and interferon-gamma levels were risk factors for severe laryngeal obstruction in children. These indicators should be concerned timely in monitoring the progression of laryngeal obstruction in children.

## 1. Introduction

Laryngitis is an acute or chronic inflammation that affects the laryngeal mucosa and submucosal tissues. Laryngitis is a secondary inflammation generally caused by viruses, bacteria, stimulants, and antigens.^[1]^ Viral infections of the upper respiratory tract are the main cause of acute laryngitis.^[2]^ However, people with allergies, malnutrition, hypersensitivity, and chronic inflammation in the upper respiratory tract, such as chronic tonsillitis, chronic sinusitis, and adenoid hypertrophy, may increase the risk of respiratory infections and laryngeal obstruction in children.^[1,2]^

Acute laryngitis are located primarily in the supraglottic region with the following pathologic findings: mucosal edema, congestion, and infiltration of inflammatory cells. The laryngeal cavity in the subglottic region becomes narrow and edematous, and secretions accumulate in the laryngeal mucus during an episode of acute laryngitis. Acute laryngitis progresses rapidly and the symptoms of laryngeal obstruction eventually develop, such as wheezing, a barking cough, and dyspnea.^[3]^ If left unchecked, laryngeal obstruction will lead to asphyxia with a variety of complications, including respiratory failure, heart failure, and disseminated intravascular coagulation, which is a life-threatening disease in children.^[3,4]^ The key for treating children with acute laryngitis and laryngeal obstruction is early detection of the clinical features and symptoms of acute laryngeal obstruction. However, few studies with a focus on the clinical characteristics that influence acute laryngitis with laryngeal obstruction have been published in children.

This study aimed to explore the clinical characteristics and factors that influence acute laryngitis with laryngeal obstruction in children and provide evidence for the diagnosis and treatment.

## 2. Patients and methods

### 2.1. Subjects and groups

A retrospective study was conducted on clinical data from 390 children with acute laryngitis who were evaluated and treated in our department from February 2021 to February 2024. The inclusion criteria included the diagnostic criteria for laryngeal obstruction^[5]^: ① sore throat, dry pharynx, pharyngeal itching, and hoarseness; ② barking cough; 3 inspiratory dyspnea with 3 concave sign; ④ congestion and swelling of the laryngeal mucosa and congestion in the vocal cords; and 5 other conditions that meet the diagnostic criteria.^[5]^ The exclusion criteria were as follows: ① children < 6 months and > 12 years of age (Nota bene, in children <6 months of age, laryngitis is often combined with other diseases, such as laryngeal malacia; in children >12 years of age laryngeal chamber development is near normal and symptoms of laryngitis resolve spontaneously); ② history of glucocorticoid intake in recent 2 months; ③ a history of laryngeal surgery; ④ congenital laryngeal, bronchial, and pulmonary diseases; ⑤ foreign body in the trachea or bronchus; ⑥ central nervous system and metabolic diseases or specific mental illness; and ⑦ severe liver, kidney, and other diseases.

This study was approved by the institutional ethics review board and oral consent was obtained from the parents of the children. Written informed consent was waived by the institutional ethical review board due to the nature of the retrospective study.

### 2.2. Methods of data collection

1. Symptoms and physical examination. Age, sex, body mass index, allergy history (dermatitis, eczema, urticarial, and asthma), course and clinical characteristics (sore throat, dry pharynx, pharyngeal itching, hoarseness, cough, expectoration, dyspnea, diffuse congestion, and swelling of the laryngeal mucosa, congestion, edema, or hypertrophy in the vocal cord region) were routinely recorded.
2. Laboratory detection data was routinely recorded. A venous blood sample (5 mL) was obtained before and after treatment in patients with acute laryngitis and laryngeal obstruction. The blood sample was centrifuged and stored at −20 °C in a refrigerator. White blood cells were counted by cytometry. The C-reactive protein level was determined using an enzyme-linked immunosorbent assay and tumor necrosis factor-alpha (TNF-α), interleukin (IL)-4, IL-6, IL-17, and interferon-gamma (IFN-γ) were determined using a flow cytometry method.
3. The data of the laryngoscopic examination included mainly diffuse congestion and swelling of the laryngeal mucosa, congestion, edema, or hypertrophy in the vocal cord region.

### 2.3. Treatment of children

Hypoxic children with indications for respiratory support therapy and oxygen inhalation were intubated. Medical treatment mainly included an intravenous injection of methylprednisolone, an intramuscular injection of dexamethasone, and an atomizing inhalation of a suspension of budesonide. The combination of an atomizing inhalation of adrenaline was needed for a ≥ III° laryngeal obstruction. The children were treated with an intravenous injection of methylprednisolone (1–2 mg/kg [maximum dose, 40 mg/dose]). Intramuscular injection of dexamethasone (H51020514, 0.3 mg/kg/dose [maximum, 15 mg/dose daily]; Tianjin Jinyao, Tianjin, China). Budesonide was inhaled after mouth washing. A budesonide suspension for inhalation (1.0 mg) was added to 2 mL of a 0.9% sodium chloride injection (H20140475; AstraZeneca Pharmaceutical Co., Ltd., North Ryde, New South Wales, Australia), then an atomization inhalation was performed with approximately 5 μm of aerosol particles 4 to 6 times a day with a 4 to 6 hours interval for 7 consecutive days.

Alternating atomizing inhalation of adrenaline. An adrenaline hydrochloride injection (H23023237, 1.0 mg; Harbin Pharmaceutical Group Sanjing Pharmaceutical Co., Ltd., Harbin, China) was added to 5 mL of a 0.9% sodium chloride injection, then atomization was performed with approximately 5 μm of aerosol particles. Atomizing inhalations were 15 minutes in duration for 3 to 5 days. Nasogastric tube feeding was administered when necessary to ensure sufficient intake of water and nutrients. The children received anti-infectives, antitussives, and expectorants, as well as back patting and sputum aspiration as needed. Children were closely monitored for disease progression. Dynamic changes in the patient’s condition were also recorded.

### 2.4. Effect evaluation

The children were divided into groups according to the diagnostic criteria for laryngeal obstruction with the corresponding symptoms,^[2]^ as follows: no laryngeal obstruction; I° laryngeal obstruction (asymptomatic when calm, while mild dyspnea and mild inspiratory laryngeal wheezing, and concave soft tissue around the chest when active); II° laryngeal obstruction (inspiratory dyspnea, inspiratory laryngeal wheezing and concave soft tissue around the chest are evident when calm, and symptoms worsen during activity but diet and sleep are not affected with no apparent hypoxic symptoms); III° laryngeal obstruction (inspiratory dyspnea and loud laryngeal wheezing, concavity in the soft tissues (suprasternal and supraclavicular windows during inhalation), dysphoria, sleep disturbances, loss of appetite, increased pulse, and elevated blood pressure); and IV° laryngeal obstruction (extreme dyspnea). The effects of acute laryngitis and laryngeal obstruction were compared in the 5 groups.

The patients were divided into 3 subgroups according to the effect of the treatment, as follows: ① significantly affected (sore throat, dry pharynx, pharyngeal itching, hoarseness, cough, and dyspnea resolved after 3 days of treatment); ② affected (sore throat, dry pharynx, pharyngeal itching, hoarseness, cough, and dyspnea were relieved); and ③ unaffected (no improvement in primary symptoms or disease progression). If the patient had blood oxygen < 90%, the patient was intubated and transferred to the ICU for mechanical ventilation.

### 2.5. Statistical analysis

The clinical data was analyzed using SPSS 25.0 statistical software. Measurement data with a normal distribution are expressed as mean differences ± standard deviation ($\bar{x}\pm s$). Data that were not normally distributed are expressed as a mean (M). A one-way analysis of variance was adopted for the overall comparison of the data in each group. The differences between and within groups were compared with LSD. A chi-square test was performed on count data, which are expressed as a percentage (%). Univariate and multivariate logistic regression analysis was used to identify risk factors and efficacy of treatment in children with acute laryngitis and laryngeal obstruction.

## 3. Results

### 3.1. General data

All the 390 children recovered well. There were 122 children in the group without laryngeal obstruction (31.88%), 125 children in the group with laryngeal obstruction I° (30.05%), 98 children in the group with laryngeal obstruction II° (25.13%), 43 children in the group with laryngeal obstruction III° (11.03%), and 2 children in the group with laryngeal obstruction IV° (0.51%). The general data of the children were shown in Table 1. Gender, age, body mass index, and the course of disease were comparable between the 5 different groups (*P* > .05).

**Table 1:** Comparison on the clinical data among different groups (n/%).

Observed Indicators	No laryngeal obstruction group (n = 122)	I° laryngeal obstruction group (n = 125)	II° laryngeal obstruction group (n = 98)	III° laryngeal obstruction group (n = 43)	IV° laryngeal obstruction group (n = 2)	Z/F	*P*
Gender (boy/girl, n)	69/53	65/60	57/41	24/19	1/1	0.199	.905
Age (years)	4.55 ± 0.45	4.25 ± 1.03	4.37 ± 0.75	4.70 ± 1.11	1.4	0.347	.749
6 months to 3 years of age	53 (43.4%)	72 (57.6%)	56 (57.1%)	25 (58.1%)	2	2.467	.036
3–6 years old (n)	39 (32.0%)	31 (24.8%)	26 (26.5%)	10 (23.3%)	0		
6–12year (n)	30 (24.6%)	22 (17.6%)	16 (16.3%)	8 (18.6%)	0		
BMI (kg/m²)	24.06 ± 3.30	23.76 ± 3.30	23.85 ± 3.21	23.78 ± 3.27	18.5	0.792	.598
Course of disease	1.82 ± 0.31	1.79 ± 0.34	1.84 ± 0.35	1.87 ± 0.42	1.9	0.469	.704
Barking cough	62 (50.8%)	75 (60%)	70 (71.4%)	35 (81.4%)	2 (100%)	24.226	<.001
Hoarseness	59 (48.4%)	71 (56.8%)	63 (64.3%)	30 (69.8%)	2 (100%)	10.257	.003
Sore throat, dry pharynx, Pharyngeal itching	55 (45.1%)	71 (56.8%)	74 (75.5%)	34 (79.1%)	2 (100%)	18.652	<.001
Wheezing	42 (34.4%)	50 (40%)	42 (42.9%)	18 (41.9%)	2 (100%)	5.218	.011
Dyspnea	41 (33.6%)	59 (47.2%)	53 (54.1%)	30 (69.8%)	2 (100%)	15.687	.001
Loss of appetite	39 (32.0%)	43 (34.4%)	34 (34.7%)	15 (34.9%)	2 (100%)	6.240	.009
Diffuse congestion and swelling of laryngeal mucosa	39 (32.0%)	59 (47.2%)	50 (51.0%)	27 (62.8%)	1 (50%)	15.204	.001
Vocal cord edema	38 (31.1%)	42 (33.6%)	36 (36.7%)	16 (37.2%)	2 (100%)	4.019	.015
Vocal cord congestion	38 (31.1%)	42 (33.6%)	55 (56.1%)	25 (58.1%)	2 (100%)	8.246	.005
Hypertrophy of vocal cord	32 (26.2%)	32 (25.6%)	27 (27.6%)	13 (30.2%)	2 (100%)	2.197	.028
History of allergies	12 (9.8%)	14 (11.2%)	11 (11.2%)	6 (14.0%)	1 (50%)	3.121	.032

Among these children, 203 cases (52%) were boys, 187 cases (48%) were girls; and a total of 44 cases (11.3%) had a history of allergies.

### 3.2. Comparison of clinical data among different groups of laryngeal obstruction

Table 1 shows the comparison of each group according to different levels of laryngeal obstruction. The morbidity rate for patients from 6 months to 3 years of age was the highest in each group and was more likely to have comorbidities, such as laryngeal obstruction (*P* = .036). The incidence of symptoms, such as sore throat, hoarseness, wheezing, barking cough, dyspnea, diffuse congestion, swelling of the laryngeal mucosa, and congestion of the vocal cord was significantly higher in the groups that were diagnosed with higher degree of laryngeal obstruction than those with lower degree (*P* < .05; Table 1).

### 3.3. Comparison of laboratory indicators in each group

The levels of WBC count, CRP, TNF-α, IL-4, IL-6, IL-17, and IFN-γ were higher in groups with high degree of laryngeal obstruction than in groups with low degree of laryngeal obstruction (*P* < .05; Table 2). The higher the degree of obstruction, the higher was the levels of the laboratory indicators.

**Table 2:** Comparison of the laboratory indicators in each group ($\bar{x}\pm \;s$).

Observed indicators	No laryngeal obstruction group (n = 122)	I° laryngeal obstruction group (n = 125)*	II° laryngeal obstruction group (n = 98)†	III° laryngeal obstruction group (n = 43)‡	IV° laryngeal obstruction group (n = 2)
WBC count (×10^9^/L)	10.06 ± 1.06	12.15 ± 1.58	13.39 ± 1.65	14.67 ± 2.30	17.25
CRP (mg/L)	4.67 ± 0.51	9.29 ± 1.62	12.99 ± 1.68	29.37 ± 3.28	79.65
TNF-α (ng/L)	105.26 ± 5.01	119.41 ± 5.69	128.76 ± 6.05	140.29 ± 7.08	239.10
IL-4 (pg/mL)	52.39 ± 6.97	81.55 ± 8.31	110.47 ± 9.20	142.88 ± 10.37	262.75
IL-6 (pg/mL)	43.58 ± 6.08	74.62 ± 7.69	108.56 ± 9.26	129.15 ± 9.30	305.15
IL-17 (pg/mL)	59.78 ± 6.97	83.96 ± 8.54	113.82 ± 9.35	147.06 ± 11.00	341.80
IFN-γ (pg/mL)	63.88 ± 6.54	90.91 ± 8.05	122.44 ± 10.24	165.08 ± 12.61	352.75

IFN-γ = interferon-gamma, IL = interleukin, TNF-α = tumor necrosis factor-alpha.

* Compared with the no laryngeal obstruction group, *P* < .05.

† Compared with the I° laryngeal obstruction group, *P* < .05.

‡ Compared with the II° laryngeal obstruction group, *P* < .05.

### 3.4. Analysis of risk factors for the effectiveness of acute laryngitis treatment combined with laryngeal obstruction

According to the standard of significant effectiveness, 263 cases had significant effectiveness and 112 cases had effectiveness. The total effectiveness rate was 96.15% (significantly effective + effective). In the univariate logistics regression analysis, effectiveness was the dependent variable, and all parameters with differences were independent variables. Independent variables included age, sore throat, dry pharynx, pharyngeal itching, hoarseness, cough, expectoration, barking cough, dyspnea, diffuse congestion, swelling of the laryngeal mucosa, vocal cord congestion, edema, hypertrophy, WBC count, and CRP, TNF-α, IL-4, IL-6, IL-17, and IFN-γ levels.

Of all parameters, age, history of allergies, clinical symptoms (including sore throat, hoarseness, wheezing, barking cough, dyspnea, diffuse congestion, swelling of the laryngeal mucosa, vocal cord congestion), laboratory indicators (WBC count, and CRP, TNF-α, IL-4, IL-6, IL-17, and IFN-γ levels) were risk factors for the efficacy of treatment in children with acute laryngitis and laryngeal obstruction (Table 3).

**Table 3:** Univariate logistic regression analysis on the efficacy of treatment in children with acute laryngitis and laryngeal obstruction.

Observed indicators	B	SE	Wald χ^2^ value	*P* value	OR value	OR (95%)
Age	0.217	0.128	3.595	.033	1.269	1.204–2.215
Sore throat, dry pharynx, pharyngeal itching	0.285	0.141	4.758	.025	1.275	1.325–2.298
Hoarseness	0.267	0.164	4.028	.038	1.272	1.274–2.226
Dyspnea	0.210	0.175	3.645	.031	1.276	1.265–2.314
Barking cough	0.298	0.128	5.247	.019	1.436	1.325–2.658
Diffuse congestion and swelling of the laryngeal mucosa	0.266	0.126	4.478	.031	1.287	1.271–2.355
Vocal cord congestion	0.452	0.169	4.217	.015	1.546	1.335–3.011
Vocal cord edema	0.426	0.164	3.988	.017	1.523	1.321–2.986
Hypertrophy of the vocal cord	0.195	0.325	0.365	.499	1.156	0.702–2.019
WBC count	0.267	0.125	4.268	.036	1.394	1.300–2.729
CRP	0.526	0.159	11.986	<.001	2.011	1.558–4.168
TNF-α	1.205	0.292	16.274	<.001	4.667	2.645–7.416
IL-4	1.224	0.199	15.242	<.001	4.589	2.569–7.208
IL-6	1.288	0.196	26.177	<.001	5.120	2.768–8.356
IL-17	1.235	0.235	13.481	<.001	4.299	2.497–6.518
IFN-γ	1.467	0.256	23.645	<.001	5.037	2.714–7.244

IFN-γ = interferon-gamma, IL = interleukin, TNF-α = tumor necrosis factor-alpha.

Multivariate logistic regression analysis indicated that TNF-α, IL-4, IL-6, IL-17, and IFN-γ were risk factors for the efficacy of treatment in children with acute laryngitis and laryngeal obstruction after adjustment for confounders, such as age and gender. All differences were statistically significant (*P* < .05; Table 4).

**Table 4:** Multivariate logistics regression analysis on the efficacy of treatment in the children with acute laryngitis and laryngeal obstruction.

Observed Indicators	B	SE	Wald χ^2^ value	*P* value	OR value	OR (95%)
TNF-α	1.304	0.190	32.487	<.001	2.965	2.105–5.654
IL-4	1.119	0.186	29.668	<.001	2.905	2.089–5.425
IL-6	1.884	0.298	34.190	<.001	3.260	2.270–6.261
IL-17	1.655	0.264	31.255	<.001	3.177	2.168–6.009
IFN-γ	1.449	0.249	29.254	<.001	3.001	2.025–5.138

IFN-γ = interferon-gamma, IL = interleukin, TNF-α = tumor necrosis factor-alpha.

## 4. Discussion

Acute laryngitis tends to occur in children more frequently with a male to female ratio of 1.5:1. The highest age incidence of acute laryngitis in children is between 6 months and 3 years of age.^[1]^ The morbidity was much higher in children 6 months to 3 years of age than in other age groups in this study, which is consistent with previous investigation.^[1,2]^ Children between 6 months and 3 years of age are more vulnerable due to the anatomic characteristics of the larynx, including the narrow laryngeal cavity and the loss of mucosal tissue in the upper airway.^[3–5]^ Slight swelling causes a 64% reduction in airway area. Irritation and crying worsen acute laryngitis.^[6]^ Additionally, the children of this age will easily have allergy.^[7,8]^ This study also has a total of 44 cases (11.3%) reported with allergies. Children between 6 months and 3 years of age who also have the history of allergies, should receive more attention.

Based on our research findings, hoarseness, throat wheezing, inspiratory dyspnea, and barking cough are common clinical symptoms of acute laryngitis and laryngeal obstruction in children. The higher is the degree of obstruction, the more significant are the symptoms in the subgroups. These results are consistent with a previous investigation.^[4–8]^ Symptoms might be the primary clinical symptoms of acute laryngitis and laryngeal obstructions in children and may be closely associated with the severity of the disease. We suggest that the clinician must pay high attention to these symptoms during diagnosis.

Acute laryngitis is mainly mediated by neutrophils. Irritation from bacteria and viruses has the ability to decrease the expression of toll-like receptor 4 in bronchial epithelial cells, activate neutrophils, and cause the organism to secrete a large number of inflammatory factors, such as IL-6 and TNF-α, resulting in a worsening of the inflammatory response and disease progression.^[1,9]^ As a type of regulatory factor for peptides, TNF-α has high biological activity and overexpression in children with laryngitis.^[9]^ IL-6 also plays a role in inflammatory reactions, anti-infections, and autoimmune responses in the human body.^[9]^ As cytokines, IL-4 and IL-17 are secreted mainly by helper T cells and are involved in high airway inflammation.^[9,10]^ IFN-γ is secreted by Th1 cells, which facilitates virus elimination and induces eosinophil infiltration.^[11]^ Huang et al^[11]^ reported that a patient with acute laryngitis has a significant elevation of IL-4, IL-17, and IFN-γ within the blood. CRP is produced by the liver and its main function is to regulate lymphocytes and increase phagocytosis. When tissues are damaged or have inflammatory stimulation due to microbial invasion, the level of CRP will increase rapidly. Therefore, CRP is one of the nonspecific markers of acute systemic inflammation that can directly reflect the activity of inflammatory tissues.^[12,13]^ According to the study by Zhang et al,^[4]^ symptoms including cough, sore throat, dyspnea, and laryngeal mucosal congestion are closely related to the severity of laryngeal obstruction. An elevated WBC count and higher levels of CRP, TNF-α, IL-6, and IL-8 represent a more severe disease.^[4]^ Our results also demonstrated that the WBC count and the increased levels of CRP, TNF-α, IL-4, IL-6, IL-17, and IFN-γ in the groups with high degree of laryngeal obstruction were significantly higher than in the groups with low degree of laryngeal obstruction. The higher the degree of obstruction, the higher was the levels of the laboratory indicators. These results were consistent with previous reports.^[9–13]^ Multivariate logistic regression analysis showed that the TNF-α, IL-4, IL-6, IL-17, and IFN-γ levels are risk factors for children with acute laryngitis combined with laryngeal obstruction.

The results in this study indicated that the increased levels of the laboratory indicators and the severe degree of clinical symptoms should be considered in a combination for the diagnosis of acute laryngitis combined with laryngeal obstruction.

Based on the study by Yang et al’s study,^[14]^ the intravenous injection of methylprednisolone and an atomized suspension of budesonide has a high efficacy rate in the treatment of children with acute laryngitis and laryngeal obstruction (up to 97.9%). Zhao et al^[15]^ concluded that alternating atomized inhalation of adrenaline and budesonide also has a high rate of efficacy in the treatment of children with acute laryngitis and laryngeal obstruction (up to 96.15% [84% with budesonide]).^[15]^ According to the study by Huang et al,^[11]^ inhaled budesonide has significant efficacy in reducing serum inflammatory factors. Our study also used similar medications for treatment. The significant efficacy group had 263 cases (67.44% [263/390]) and the efficacy group had 112 cases (28.71% [112/390]). Therefore, the clinical plan was shown to be effective. Fifteen cases were ineffective. Children with blood oxygen < 90% who were intubated were transferred to the ICU for mechanical ventilation. These 15 cases were ultimately recovered. For children with laryngitis complicated by severe pneumonia, early medical intervention is necessary to control respiratory inflammation. When the status is severe, the treatment method must be changed immediately.

There were several limitations in this study. This was a single-center-based study which may need further investigation based on a multicenter study in the future. There was a lack of analysis in patients with different medication plans. The impact of different medication plans on the symptoms of children has not been fully explored.

In conclusion, the incidence of sore throat, hoarseness, wheezing, barking cough, dyspnea, diffuse congestion and swelling of the laryngeal mucosa, and vocal cord congestion is highly associated with children between 6 months and 3 years of age, a history of allergies, and the severity of laryngeal obstruction. Concerns about these indicators should be addressed in clinical practice.

## Author contributions

**Conceptualization:** Yueting Zhu, Xuyuan Sun.

**Data curation:** Yueting Zhu, Xuyuan Sun, Guangping Liu.

**Formal analysis:** Yueting Zhu, Xuyuan Sun, Guangping Liu.

**Funding acquisition:** Guangping Liu.

**Methodology:** Yueting Zhu, Xuyuan Sun, Guangping Liu.

**Project administration:** Yueting Zhu, Xuyuan Sun.

**Writing – review & editing:** Yueting Zhu, Xuyuan Sun, Guangping Liu.

**Writing – original draft:** Guangping Liu.
